# Process evaluation of appreciative inquiry to translate pain management evidence into pediatric nursing practice

**DOI:** 10.1186/1748-5908-5-90

**Published:** 2010-11-20

**Authors:** Tricia Kavanagh, Bonnie Stevens, Kate Seers, Souraya Sidani, Judy Watt-Watson

**Affiliations:** 1 Lawrence S. Bloomberg Faculty of Nursing, University of Toronto, Toronto, Ontario, Canada; 2 The Hospital for Sick Children, Toronto, Ontario, Canada; 3 Faculty of Medicine, University of Toronto, Toronto, Ontario, Canada; 4 RCN Research Institute, School of Health & Social Studies, University of Warwick, Coventry, UK; 5 School of Nursing, Ryerson University, Toronto, Ontario, Canada

## Abstract

**Background:**

Appreciative inquiry (AI) is an innovative knowledge translation (KT) intervention that is compatible with the Promoting Action on Research in Health Services (PARiHS) framework. This study explored the innovative use of AI as a theoretically based KT intervention applied to a clinical issue in an inpatient pediatric care setting. The implementation of AI was explored in terms of its acceptability, fidelity, and feasibility as a KT intervention in pain management.

**Methods:**

A mixed-methods case study design was used. The case was a surgical unit in a pediatric academic-affiliated hospital. The sample consisted of nurses in leadership positions and staff nurses interested in the study. Data on the AI intervention implementation were collected by digitally recording the AI sessions, maintaining logs, and conducting individual semistructured interviews. Data were analysed using qualitative and quantitative content analyses and descriptive statistics. Findings were triangulated in the discussion.

**Results:**

Three nurse leaders and nine staff members participated in the study. Participants were generally satisfied with the intervention, which consisted of four 3-hour, interactive AI sessions delivered over two weeks to promote change based on positive examples of pain management in the unit and staff implementation of an action plan. The AI sessions were delivered with high fidelity and 11 of 12 participants attended all four sessions, where they developed an action plan to enhance evidence-based pain assessment documentation. Participants labeled AI a 'refreshing approach to change' because it was positive, democratic, and built on existing practices. Several barriers affected their implementation of the action plan, including a context of change overload, logistics, busyness, and a lack of organised follow-up.

**Conclusions:**

Results of this case study supported the acceptability, fidelity, and feasibility of AI as a KT intervention in pain management. The AI intervention requires minor refinements (*e.g.*, incorporating continued follow-up meetings) to enhance its clinical utility and sustainability. The implementation process and effectiveness of the modified AI intervention require evaluation in a larger multisite study.

## Background

Knowledge translation (KT) is broadly defined as 'a dynamic and iterative process that includes synthesis, dissemination, exchange, and ethically-sound application of knowledge to improve the health of Canadians, provide more effective health services and products, and strengthen the health care system' [1]. Translating evidence into practice is a complex, multifaceted process, yet there is a lack of clarity around which interventions are effective, with whom, and in what contexts [2]. Reviews of interventions to implement clinical practice guidelines in healthcare indicate that they are variably effective in different contexts [*e.g.*, [3-5]]. In light of this complexity, theory has been implicated as important to designing and evaluating KT interventions [6-8].

Appreciative inquiry (AI) is a promising theory-based KT intervention that is compatible with the Promoting Action on Research in Health Services (PARiHS) framework [2,9,10]. With roots in organisational change and action research, AI has a unique focus on existing organisational strengths, rather than weaknesses, to enhance practices [11]. The AI process consists of the 4-D cycle: Discovery (positive elements of practice are illuminated), Dream (an ideal practice environment is envisioned), Design (processes are created that support the ideal), and Destiny (strategies are implemented that strive for the ideal) [11]. The theoretical relevance of AI as a KT intervention applied to the clinical issue of pain has been proposed [12].

Essentially, AI can be conceptualised as an enabling process of facilitation, with the potential to address the nature of the evidence and context in which evidence is to be implemented to promote evidence-based practices in healthcare [12].

Although AI holds theoretical promise as a KT intervention, it has yet to be applied or evaluated as such. AI has been largely used to enhance administrative- or human-resource-related topics in the business [*e.g.*, [13-15]] and healthcare literature [*e.g.*, [16-18]]. Exploratory studies are recommended to select and refine KT interventions in clinical healthcare [6]. Pilot work examining feasibility is an important first step to developing and evaluating complex interventions [19], and process evaluations are considered essential to gaining insight into why and how complex interventions work to optimize them for future evaluations [20].

In this paper, the main findings regarding the implementation of AI as a KT intervention in pain management are presented. Exploration of the AI intervention implementation in this theoretically based study specifically sought to examine the acceptability, fidelity, and feasibility of using AI to implement pain management evidence in pediatric nursing practice to support its refinement for future evaluation in a larger-scale study. Although pain is an interprofessional responsibility, nurses were the focus in this study given their pivotal role in pain management [21] and the exploratory nature of the study design.

### Study objectives

The primary objective of this study was to determine the acceptability, fidelity, and feasibility of the AI intervention. Acceptability is the suitability of the intervention from the perspectives of the participants [22] and was operationalised in terms of nurse participants' perceived relevance of the AI intervention for translating pain management evidence into practice. Fidelity is the extent to which the intervention could be delivered as intended [22] and was operationalised as the consistency of its implementation with the essential elements of the AI process and nurse participants' perceptions of barriers to its implementation. Feasibility is the ease of executing the intervention [22] and was operationalised in terms of maintaining nurse participants' attendance at AI sessions, completing the phases of the AI process in four 3-hour sessions, maintaining the content focus of the AI sessions on pain management evidence, and the frequency and duration of the AI sessions needed to reach all nurse participants.

## Methods

A mixed-methods case study design with convergent triangulation was used. The case was a unit within a hospital. Quantitative and qualitative data were collected concurrently to gain broader perspectives on the research questions and integrated in the discussion to add depth to the interpretation of the findings [23].

### Setting and sampling technique

The study setting was a 25-bed surgical unit at a university-affiliated pediatric hospital in Canada. The AI intervention sessions were delivered in hospital meeting rooms. Purposive sampling was used to select nurse leaders in administrative, clinical, and educational roles, and convenience sampling was used to select all staff nurses interested in participating. Students and nurses intending to terminate their positions in the unit during the study period were ineligible. There were 54 staff nurses and three nurse leaders in the study unit at the time of recruitment.

### AI intervention

The AI intervention consisted of two components: staff participation in four facilitator-led sessions based on the 4-D cycle [11] of the AI process and staff implementation of an action plan to enhance evidence-based pain practices in their unit, as generated in the last AI session. Each AI session was three hours long and delivered over two weeks (Table 1). The AI sessions were centered on the broad affirmative topic: What is working well for practicing evidence-based pain management in your unit? Participants selected the specific topic of evidence-based pain assessment documentation in the Dream phase based on a desire to enhance the quality of documentation practices in their unit. With facilitator support, the participants ultimately developed a contextually tailored action plan, which included audit and feedback with education (Table 2); they implemented the plan independently over approximately two months following attendance at the AI sessions. The lead author (Process Facilitator) and a Master's-prepared nurse practitioner from the hospital's Acute Pain Service (Content Facilitator) codelivered the AI sessions based on their knowledge of AI and pain, respectively. A postdoctoral student with expertise in pediatric pain and KT was a back-up facilitator, who mainly acted as a recorder during the AI sessions. The lead author developed an intervention manual that provided specific directions for the facilitators to implement the essential elements of the AI process. Participants were compensated with Can$400 for completing all of the AI sessions, as staff nurses were required to attend the sessions on scheduled days off.

**Table 1:** Summary of the AI sessions

	Discovery	Dream	Design	Destiny
Purpose	To focus on positive examples of using pain management evidence in practice	To envisionan ideal context for using pain management evidence in practice	To create contextual structures and processes that support the ideal for using pain management evidence in practice	To implement contextually tailored strategies that strive for the ideal for using pain management evidence in practice
				
Activities	Introduction to the AI process; explanation of 'high' evidence applied to pediatric pain management; reframing evidence-based pain management as an Affirmative (or positively phrased) Topic; engagement in appreciative interviews to explore positive examples of evidence-based pain management	Consideration of Miracle Questions or questions to envision the possibilities and related contextual supports for using pain management evidence in everyday practice; selection of a specific topic	Formulation of a collective Provocative Proposition or a realistic, present tense, affirmative statement outlining the possibilities for using pain management evidence in everyday practice	Creation of a contextually tailored, concrete action plan to implement pain management evidence in everyday practice within a three-month period
				
Frequency and duration of sessions	One 3-hour session delivered in a two-week period	One 3-hour session delivered in a two-week period	One 3-hour session delivered in a two-week period	One 3-hour session delivered in a two-week period

AI = appreciative inquiry

**Table 2:** Summary of the action plan

Action Item	Description
1	Create and display a poster of the Provocative Proposition, as developed during the Design phase
	
2	Develop and implement a self-learning module for all nurses to complete, based on the hospital clinical practice guideline for pain assessment and documentation
	
3	Implement positive, nurse-to-nurse, same-day audit and feedback to promote evidence-based pain assessment documentation by all nurses in the unit, based on the hospital clinical practice guideline for pain assessment and documentation

### Data collection

**Figure 1:**
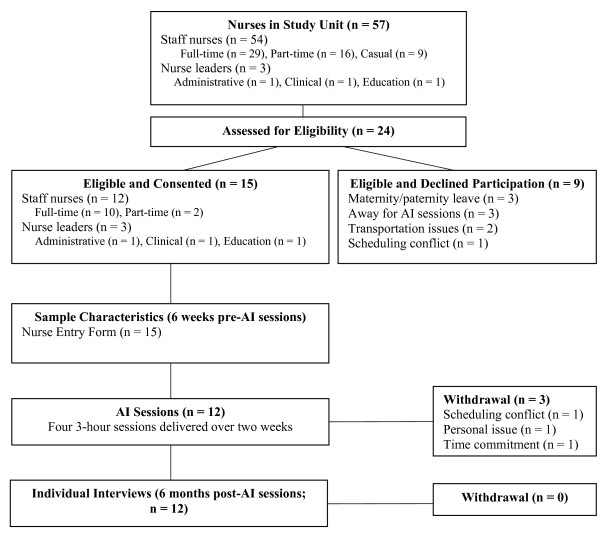
**Study schema**. Study schema outlining the derivation of the sample, data collection, and the AI intervention. AI = appreciative inquiry.

Following Research Ethics Board approval and informed consent, baseline demographic data for nurse participants were obtained using the Nurse Entry Form developed by the lead author. Acceptability and fidelity data for the AI intervention were collected by a research assistant (otherwise unaffiliated with the study), who conducted individual face-to-face semistructured interviews with all participants regarding their views on AI as a KT intervention and barriers to their participation in the AI sessions and implementation of the action plan. The AI process was distinguished from the AI sessions in the interview guide, where *process *referred to the broad theory and principles underlying the 4-D cycle (*e.g.*, positive, participatory, organisational focus) and *AI sessions *consisted of the concrete activities and structural elements (*e.g.*, number and duration of sessions, group characteristics, roles of the Process and Content Facilitators) used to bring the AI process into practice for the purpose of the study. The interviews were conducted six months after the delivery of the AI sessions to allow the participants sufficient time to implement the action plan in their unit and provide a preliminary exploration of sustainability (Figure 1). All interviews were digitally recorded, with consent, and lasted from 30 to 60 minutes. Individual interviews were used because it was thought that staff nurses may have limited the extent of their disclosure in a focus group due to the presence of nurse leaders, and surveys may not have provided the desired depth of feedback. Fidelity of the intervention was also assessed by digitally recording the AI sessions for comparison with the intervention manual. Feasibility of the AI intervention was measured by recording participants' reasons for declining participation; documenting their attendance at the AI sessions in a Group Log; documenting the frequency and duration of the delivered AI sessions, defined by the total number of times each AI session was delivered in a given time period and the number of minutes per session, respectively, in the Facilitator Log; and recording the total duration, in weeks, of the AI sessions in the Facilitator Log. Participant confidentiality was maintained by assigning each nurse participant a study code number to identify questionnaires. Completed data forms were kept in a locked filing cabinet in the lead investigator's office and access to data on the computer was password protected and encrypted to comply with current privacy legislation.

### Data analysis

Descriptive statistics were used to analyse quantitative data related to the sample. Qualitative content analysis [24-26] was conducted on verbatim transcripts of the semistructured interviews by the lead author to determine the acceptability and fidelity of the AI intervention. Concepts were derived inductively from the data using open coding [24] and assimilated into a conceptual index of main themes and subthemes [25]. NVivo 8 was used to manage the data. Memos were written to maintain a record of concept development and analytic decisions, and a reflexive journal was kept to record reactions to the data and examine biases. A second analyst independently coded two transcripts using the conceptual index. In the case of discrepancies, resolutions included maintaining the original language for and meaning of a concept, changing the language used for a concept to more accurately reflect the meaning of a phenomenon, or adding a new concept to more comprehensively reflect the content of the data.

Quantitative content analysis was conducted on verbatim transcripts of the digitally recorded AI sessions for comparison with a template derived from the intervention manual to determine the consistency of the implemented AI sessions with the elements of the 4-D cycle of the AI process and the feasibility of the Content Facilitator maintaining a focus on pain management evidence. In both cases, the total number of activities missed out of those designed was counted. The length of time, in minutes, taken to complete each phase of the 4-D cycle was derived from the digital tapes and confirmed with the Facilitator Log. In terms of feasibility, the sample was described with respect to nurse participants' attendance at each of the four 3-hour AI sessions, the number of participants recruited and declined, and reasons for nonparticipation. Descriptive statistics were used to determine the frequency with which each AI session was delivered; the duration of each AI session delivered compared to the planned duration, in minutes; and the total duration of the AI sessions delivered, in weeks.

## Results

### Sample characteristics

A total of 24 nurses were interested and eligible to participate in the study; 12 (9 staff nurses; 3 nurse leaders in administrative, clinical, and education roles) participated, 3 consented and withdrew, and 9 decided not to participate due to personal or logistical reasons (Figure 1). The majority of participants were staff nurses, female, and employed in full-time positions in the study unit. Half of the participants were diploma-prepared and most (n = 8) had greater than six years of nursing experience. Employment duration varied, ranging from 6 months to 25.17 years (median = 7.96 years). Characteristics of the nurse participants are summarized in Table 3.

**Table 3:** Nurse participant characteristics

Characteristic	Number (%)* (n = 12)
Sex	
Female	11 (91.67)
Male	1 (8.33)
	
Employment duration in the acute care unit (months),	
Median (IQR)	95.50 (177.50)
	
Experience in nursing (years)	
0-2 years	3 (25.00)
2.1-6 years	1 (8.33)
>6 years	8 (66.67)
	
Employment position in the acute care unit	
Staff Nurse	9 (75.00)
Nurse Leader	3 (25.00)
	
Highest level of nursing education	
Diploma	6 (50.00)
Baccalaureate	4 (33.33)
Master's	2 (16.67)
	
Employment type in the acute care unit	
Full-time	10 (83.33)
Part-time	2 (16.67)
	
Pain conferences attended since basic nursing degree	
0	7 (58.33)
1-3	3 (25.00)
>3	2 (16.67)

*Percentages within characteristics may not add to 100% due to rounding.

IQR = interquartile range.

### Acceptability of the AI intervention

Participants discussed aspects of the AI intervention that they liked and areas for improvement related to both the AI process and AI sessions.

#### Views on the AI process: A refreshing approach to change

Participants liked the AI process, enjoyed participating in it, and found it a valuable way to approach practice change. The AI process was considered distinct from typical change initiatives and appealing in its atypicality:

It's usually, 'here's what we're working with, what can we change' as opposed to 'this is what you guys are doing and doing well, how can we expand and make it better...than what it already is'. It was actually for a lot of us, I think it was quite exciting to have this sort of study being done as opposed to the usual ones that we do. (Interview 09, p. 1, lines 22-25)

Some participants indicated that they would readily participate in another AI intervention or that it would be fitting for other interventionists to assume an AI approach. AI was considered a clinically useful intervention because it was applicable to other areas besides pain. It was characterized as a refreshing approach to change due to its positive approach, democratic nature, and focus on expanding on existing practices.

#### The positive approach of the AI process

It's good in the way that it acknowledges what we're doing right and the strengths that we have and then it just helps us to strengthen whatever it is that we're already doing well into something better, and I really like that part of the whole process. (Interview 05, p. 1, lines 12-14)

Participants repeatedly praised the positive approach of the AI process, which included giving attention to strengths and successes in their unit related to pain and other clinical areas. Engagement in AI was described as rewarding, motivating, and empowering. Although the group liked holding a positive focus through the AI sessions, this task was not necessarily felt to be effortless; it was perceived as a novel approach in a context (*i.e.*, society and work environment) that was more attentive to the negative. Acknowledging issues and challenges was considered important to avoiding negative sentiments around maintaining a strictly positive focus:

Like even though we were talking positive, positive, positive but we were looking at all the negative aspects and trying to make that positive. So I don't think that anybody in the group actually felt anything different or felt negative about only talking about positive and not the negative aspect of what we do on the floor. (Interview 08, p. 2, lines 6-9)

#### The democratic nature of the AI process

There was widespread enthusiasm about the democratic nature of the AI process amongst participants, but especially from the staff nurses. Staff nurse participants often contrasted the AI process to the more dictatorial approaches to change (speaking explicitly about being 'dictated to') that they were accustomed to in the unit:

I don't know of any other [approaches to change] other than being sort of told what we should do. And this was a nice, refreshing approach to collecting information. I think it worked well because like I said, I was very impressed with it because I guess a lot of times when we're the ones that are actually doing the work, we're not the ones that are asked questions about what we should be doing or how we should do it-we're being told what we should do, right? And it's nice to be able to give the input because a lot of us, like I said have many years of experience and knowledge behind this stuff...and it does support, you know, the changes, you know? (Interview 06, p. 6, lines 28-45)

Staff nurse participants discussed their appreciation of being involved in the AI intervention from the outset and the equal participation of staff nurses and nurse leaders alike. Being leaders of the change was relished, and the experience of working together as equals in a group was described as fun, exciting, and rewarding. Implementing the action plan in their unit without outside assistance was considered empowering; overall, a continued relationship with the facilitators was not desired, as participants felt they had enough support amongst themselves to enact the plan. The nurse leaders spoke of the benefit of involving staff nurses in the change initiative, including the value of gaining contributions from those who would use the practice, their ideal position in the unit to defend the change to their colleagues, and the positive influence on their professional esteem.

Despite the increased workload associated with this approach, some of the staff nurse participants remarked that it felt less burdensome relative to more dictatorial initiatives; the load of change was lightened by the fun associated with their involvement in the initiative, not being told what to do and how to do it, and working with their colleagues and the nurse leaders. However, one of the novice staff nurse participants noted that the responsibility of implementing the plan was challenging to manage due to time constraints. She used protected time from another role she assumed in the unit to implement her audits and felt that, although it was likely not practical and might be unacceptable to others, implementing the action plan outside of work time might be easier.

#### A focus on expanding on existing practices

Expanding or improving on existing unit practices, rather than implementing something entirely new, was viewed as a practical and realistic way to approach change. Overall, participants noted that expanding on existing practices eased and supported their implementation of the action plan as an independent group; they were already doing the practice and were therefore confident about the change they were putting forth. However, another participant noted disappointment around the topic choice of pain assessment documentation for this very reason, stating that it 'wasn't a far stretch to implement it on the unit' (Interview 02, p. 3, line 5). The prospect of implementing a new practice, while not impossible, was seen to be a bigger challenge that could be facilitated by the positive approach:

I think the biggest, the most key thing in this whole study was that it was an actual positive approach. It was...no matter what it was or how familiar we were with it or unfamiliar or how new or old, I don't think that matters. I think the fact that we've taken something that we're already doing whether it's something fairly new or something that we've, you know...done forever, taking that and just expanding that no matter how big or how little, I think it's that positive approach to change that makes the difference. (Interview 09, p. 6, lines 27-32)

The AI process was also considered a means to build on existing *ways *of practicing in the unit. Participants purposefully developed pain assessment documentation audits that were delivered colleague-to-colleague. Informal interactions with their colleagues were considered a natural and usual way of addressing practices in their unit. As one participant said, 'Just talking about improving practices and that kind of thing, like we do it everyday' (Interview 05, p. 13, lines 18-19).

#### Views on the AI sessions

Participants' views on the AI sessions were organised into three themes, including the structure of the sessions (*i.e.*, number, frequency, and duration), nature of the group (*i.e.*, group size, mix, and dynamics), and facilitator partnership.

#### Structure of the sessions

Overall, participants liked the number, frequency, and duration of the AI sessions. The duration of the AI sessions was cited as generally satisfactory and an important element of the intervention design, with one participant stating, 'I felt comfortable sharing my thoughts and views and I don't think that would have been possible if it felt very rushed' (Interview 07, p. 15, lines 32-34). An exception was the AI session addressing the Design phase, which participants felt required more time due to the nature of the activity; everybody had contributions to the Provocative Proposition (Table 1), and the group was intent on creating a statement that was an accurate reflection of their thoughts and intentions. Participants suggested that a practical solution to accommodate the need for more time was to add an AI session, rather than lengthening each one.

There was general disagreement around the acceptability of the full-day AI session that covered the Discovery phase in the morning and the Dream phase in the afternoon. Some participants thought it was a good day because, 'It focused on what we did well and wanted to do better' (Interview 05, p. 8, line 16); they felt the material was fresh in their minds, and they liked reducing the number of session days. More commonly, however, participants found it to be a long day, tiring, and not as productive as a result. The nurse leaders found the full day to be too long because they were also working during the AI sessions.

Keeping the sessions closely spaced was considered essential to maximizing continuity and minimizing disassociation from the content and process of the AI sessions. Emphasis was placed on the cumulative nature of the AI sessions. Overall, participants indicated that they liked completing the AI sessions within a two-week period and felt that decreasing the frequency to even one session per week might make it too long and compromise their productivity. However, there was a tension between the theoretical preference for closely spaced sessions and the practical realities imposed by the work environment:

[The spacing of the sessions] was good that way because it didn't...we didn't have much time between each session which was the good part because all the stuff that we talked about in the session before, it was quite fresh in our minds. I think if we had done once a week it would have taken us a little bit longer to get back to where we were...when we did the previous one. On the other hand, having them that close together is hard because you have to do it on your days off. And it's hard to get...I mean it's a pretty big group and it's hard to get everybody off at the same time without compromising...the unit. (Interview 09, p. 15, lines 13-22)

#### Nature of the group

Overall, participants were satisfied with the size of the group. A fine balance was noted between group size and productivity, with a recurrent view that the size was at its maximum in terms of effectiveness: More people would have meant more opinions, which might have become unmanageable. Based on the plethora of opinions expressed during the AI sessions, one participant felt that the group size was too large. She acknowledged that the larger group was helpful for implementing the action plan but that a smaller group could have selected a smaller area for change. However, it was more commonly noted that there was strength in numbers, which was important for bringing the change to the unit.

And they knew quite a few of us were interested in it so I think having us act as leaders and being involved and interested, it showed that 'why are they interested in that? Well maybe I should be too.' And I don't know, I think it really...that sort of thing works well on our unit - just having the numbers sort of speak for themselves. (Interview 12, p. 8, lines 44-46; p. 9, lines 1-3)

The value of the relatively large group size was often discussed in the context of group mix. The diversity of experiences and professional roles in the group was considered an asset to the AI sessions and potentially compromised by involving fewer participants. Several participants noted that the group dynamic was one of equality with open communication. Techniques used by the Process Facilitator were felt to promote this dynamic, including individual, paired, and group approaches to activities and addressing the quieter participants by name. Staff nurses highlighted the value of the positive focus for easing discussion around their practices and unit in the presence of nurse leaders:

And the way that everybody framed the sentences also was again to reflect more the positive than the negative because as [the Process Facilitator] kept on saying...'think about the positive aspects, we are not here for the negative ones'. So that again influenced the way we brought information out to the table without having to fear that my [nurse leader] is sitting here or my [other nurse leader] is sitting here. (Interview 08, p. 14, lines 19-23)

#### Facilitator partnership

The partnering of the Process and Content Facilitators and their distinct roles were emphasised as being essential to the AI sessions. An important aspect of the Process Facilitator's role was her provision of theory-based information on the AI process in simple language. The Content Facilitator was viewed as contributing pain-related information and, as one participant articulated, 'a practical sense of what we do on the unit' (Interview 10, p. 22, line 5). Their partnership was valued because they contributed different perspectives, ideas, and experiences to the group. Their good and complementary relationship was considered influential to group functioning and the prevention of conflict.

In light of the group size, one participant noted the value of having a back-up facilitator who could focus on recording the results generated in the group discussions. Recording results on large sheets of paper in real time was considered a valuable design feature of the AI sessions as it facilitated the development of ideas, focused the group, provided reminders of material covered, and gave an overview of the contributions of the team. Other facilitator-led features of the AI sessions that participants felt enhanced productivity were the Process Facilitator providing summaries of the activities before the sessions and handing out synopses of the discussion points from the previous session to start the next session.

### Fidelity of the AI intervention

#### Consistency of intervention implementation with the elements of the AI process

The Process Facilitator delivered all 23 activities (100%) outlined in the intervention manual as designed over the four 3-hour AI sessions. Beyond delivering the essential elements, the Process Facilitator repeated and clarified explanations and instructions around the AI process, answered participants' questions related to AI, and facilitated the development of ideas.

#### Nurse participants' perceptions of the factors that interfered with intervention implementation

Participants described several barriers that adversely affected their participation in the AI sessions and the implementation of the action plan in the unit, including change overload, logistics, busyness, and a lack of organised follow-up. There was often a divide in perspectives on barriers between the staff nurses and nurse leaders. Overall, participants stated the implementation of the action plan was a discrete event limited to the outlined tasks that was implemented in full and as planned.

#### Change overload

The thing is when we were trying to implement it, it was a really tough time because there were so many things on the unit that were changing...[the] IV pumps, the whole change of the computer system. It was just everyone was going through change overload. (Interview 05, p. 6, lines 1-3)

A context of change in the unit during the implementation of the action plan was attributed to several concurrent hospital initiatives, including the introduction of new intravenous pumps and a computer system, as well as staff nurse orientees. While some staff nurse participants indicated they felt no effect of the hospital initiatives on the implementation process, the widespread sentiment was that they slowed their progress; however, this was largely attributed to the impact of the changes on a nurse leader, rather than on themselves:

And I think that's where we ran into that issue about not being able to get our [education module]...the email sent out on time...because whoever was doing that was dealing with IV pumps and it was just ...it was a bit too much from that end I think but from our end because we weren't all...all of us were not that involved with the IV pumps, I think you know if we got the email out we would have been able to stick to [the timeline]. (Interview 09, p. 24, lines 13-17)

In spite of this transient context of change, participants noted that the long-standing culture in the unit was one of 'passion for pain management'. In general, they felt this culture facilitated their participation in the intervention sessions and supported their implementation of the action plan in the face of contextual barriers. Other cultural features outside of pain considered to make their unit a favorable setting for the AI intervention included a sense of curiosity in the unit around new initiatives consequent to it being a teaching hospital; the fact that it was a 'fairly young unit, a kid's hospital, we like to have fun and stuff like that, and people are fairly positive on the unit anyways' (Interview 02, p. 13, lines 26-27); a dynamic of equality and teamwork; and a sense of autonomy amongst the staff nurses.

#### Logistics

Organisational details, like summer holidays, were cited as interfering with the implementation of the action plan. Staff nurse participants mainly discussed the effects of a delay resulting from a nurse leader delivering late on an early phase of the action plan. This caused mild frustration on the part of some staff nurses, who felt it decreased their momentum. Others expressed understanding that the delay was a function of the nurse leader's workload, which was compounded by the unexpected leave of a participant meant to be her support for the task. One staff nurse participant noted that this delay was a judicious decision given the context of change:

There were so many things all at the same time... that I think that's why [nurse leader] decided to hold back ... because otherwise you do get, you know people not doing it...there's not compliance, they don't care, you know it's just too much all at one time, yeah. (Interview 06, p. 23, lines 7-9)

Ultimately, some staff nurses reported that they pushed forward with the plan in spite of this delay to stay on target with their deadlines. Conversely, the nurse leaders tended to focus on the logistical barriers of their professional roles and practice. They indicated that the structure of their schedules and nature of their responsibilities made it difficult to free up the time for the AI sessions. For example, one nurse leader noted,

From my perspective it was kind of hard to be away from what I had to do because it was different...like for the staff nurses it was actually off-days. So they came in on an off-day to do it...where as I would have to leave my stuff, my duties for that day to go and be away for a period...I couldn't stay for the whole [full-day session]. I had to leave for a bit of it. Because it was part of my workday and it was just...I tried to see if I could free myself up for that time but I couldn't. (Interview 10, p. 8, lines 39-42; p. 9, lines 25-26)

They discussed the inconsistency of their participation with some frustration, and one nurse leader emphasized that it was unfair to the staff participants. A staff nurse participant echoed this sentiment and felt that all participants should be expected to maintain an equal and full level of participation in the AI sessions.

#### Busyness

Participants' discussed their perceptions of juggling their work with the implementation of the action plan, within the time limits of their day. In general, staff nurse and nurse leader participants differed in their views related to this theme. Some staff nurses mentioned the adverse impact of a busy day on their efforts to complete their audits, as patient care was the priority of their daily work. Overall, however, the work of the action plan was considered feasible due to its concrete and realistic nature. The 'doable' nature of the action items and deadlines facilitated the timely implementation of the plan, despite their clinical demands. They achieved their goals by consciously including them in their daily work:

I think we find a way of just implementing it as part of our daily routine. And once you get organised and you know that that's what you're gonna do...and you put it down there, like it's on your worksheet and it's on your...[daily agenda]. (Interview 03, p. 21, lines 15-19)

The availability and accessibility of pain management resources helped their efforts, including the pain service, pain assessment tools, and pain policies and guidelines. Human resources were considered a valuable support to their practices; colleagues were a trusted source of and expedient means to information in light of their daily busyness.

Conversely, the nurse leaders noted a stronger effect of everyday busyness on their efforts to implement the action plan. Amidst juggling their administrative or clinical tasks, the implementation process was discussed as challenging. As one nurse leader stated,

I know I didn't get to all the [audits]; I was supposed to do it and it was just other...other...priorities that got in the way...Just busy, you know just everyday like stuff going on the floor and whether or not I took time so then I kept thinking 'well I should do it, I should do it' and then I just never did it and forgot about it. (Interview 11, p. 19, lines 10-11; p. 20, lines 4-6)

#### Lack of organised follow-up

The lack of organised follow-up postimplementation of the action plan was recurrently discussed by participants as impeding their continued efforts to improve pain assessment documentation in their unit. They desired a group discussion around what was implemented and how it worked, which would also have provided a conclusion:

I think we're missing that part...what's happened after you had the audits and what came out of it. Like to go back and just give feedback as to what people [felt] came about in their little, you know practices that they had to do on the unit so that everybody feels like there is some sort of closure, yeah. (Interview 03, p. 12, lines 19-22)

In the final remarks of the last AI session, the Process Facilitator emphasized that the group was to implement the action plan in their unit and use AI to continue to improve this practice area or other areas of interest. Positive momentum for change is a theoretical outcome of participating in the AI process and an aspect of creating an appreciative learning culture [11]; however, there was notable confusion amongst participants regarding who was responsible for organising a follow-up discussion. As stated by one nurse leader,

I think that maybe if we'd had another opportunity to go back as a group, that might have helped just keep the momentum going. And I don't know whether that's something that maybe the [other nurse leader] and I should have done formally or we should have utilised [the facilitators] to help with that, I'm not sure...but I think that would have helped. (Interview 11, p. 2, lines 44-45; p. 3, lines 1-2)

This confusion was linked to the democratic approach of the AI process: Because the group dynamic in the AI sessions was one of equality, when the group went forward without the guidance of the facilitators, there were no identified leaders to assume organisational roles and direct the progression of the practice change. Despite their preference for implementing the action plan without continued facilitator involvement, several participants indicated that they were relying on the facilitators to organise a follow-up meeting, rather than taking charge of the situation as a group.

### Feasibility

#### Maintaining the participants' attendance at the four 3-hour AI sessions

The majority of participants (n = 11) attended all four AI sessions, with the exception of one nurse leader who missed the last session (Destiny) due to personal reasons. There was a pattern for nurse leaders to arrive late, leave early, or come in and out of the AI sessions; however, none of the participants missed key elements or content addressed in the sessions.

#### Completing the AI process in four 3-hour AI sessions

The length of each AI session was 180 minutes (3 hours), with the 4-D cycle of the AI process completed within a total of 720 minutes (12 hours); however, completing the Dream and Design phases required more time than anticipated, and activities for these phases 'spilled over' into their subsequent AI sessions. A comparison of estimated and actual completion times for each phase of the AI process is presented in Table 4. The Dream phase was longer than expected due to the volume of contributions around the Miracle Questions (Table 1) and topic selection. The Design phase was lengthened by explanations, development, and discussions about the Provocative Proposition (Table 1). The development of the action plan was consequently shortened in the Destiny phase, which did not appear to impact its timely completion.

**Table 4:** Time requirements for each AI phase

AI Phase	Estimated Time (minutes)	Actual Time (minutes)	Difference Between Estimated and Actual Times (minutes)
Discovery	180	180	0
Dream	180	210	+30
Design	180	205	+25
Destiny	180	125	-55

AI = appreciative inquiry.

#### Maintaining the content focus of the AI sessions on pain management evidence

The Content Facilitator delivered all 12 activities (100%) as designed in the intervention manual over the four 3-hour AI sessions and maintained a focus on pain management evidence. Beyond delivering the essential elements, the Content Facilitator answered participants' questions relating to pain and facilitated the development of ideas.

#### Number of times each AI session was offered and total duration of the AI sessions

Each of the four AI sessions was offered and delivered once over two weeks. The Discovery and Dream phases were held on the first day, the Design phase was delivered three days later in the same week, and the Destiny phase occurred seven days later.

## Discussion

### Implementation process of the AI intervention

Overall, the AI intervention was implemented with high fidelity, was well accepted by participants, and was considered feasible for use as a KT intervention for pain management in an inpatient clinical setting. Participants acknowledged the positive and democratic nature of the AI process, where existing strengths, resources, and practices were used to promote practice change in contrast to the usual focus in pain on problem-focused, didactic education and/or individual persuasion interventions [*e.g.*, [27,28]]. Ultimately, the AI intervention appeared to provide a practical and appealing way to meet recommendations that KT interventions tap into human sources of knowledge, maximize interactivity, and be contextually sensitive [29,30].

Although change overload, busyness, logistics, and a lack of organised follow-up were described as barriers to the fidelity of the intervention, they were not 'critical fail factors' [20] in terms of participants' overall attendance at the AI sessions or their implementation of the action plan in a timely manner. The context (*e.g.*, resources) and culture of the study unit appeared conducive to the AI intervention and may have been important moderating factors to overcoming these barriers. Notably, a lack of organised follow-up was identified as a significant impediment to participants' sustained motivation and progression with practice enhancements in the unit. Facilitation may have an important role in improving outcomes in implementation research, especially in the face of contextual challenges [31,32]. Despite its conceptual relevance [33], a sustained external facilitator relationship was not operationalised in this study for pragmatic reasons. Capitalizing on the local human resources to facilitate long-term changes may be a way to promote and sustain interventions, where local champions are identified and trained to carry forward with the implementation [31,32,34]. Moreover, scheduling regular meetings for feedback in the action plan and outlining a long-term evaluation plan tailored to the KT strategies designed by participants may be important [31,32]. Incorporating these elements may improve the adaptability of the KT strategies generated through the AI sessions or their capacity to survive in the absence of external facilitators or presence of organisational changes [35]. Adaptability is essential to sustainability [35], and the AI process may have particular benefit in this regard, as it builds on what exists and participants can incorporate contextual changes into their action plan over time.

### Implications for future evaluations of AI

Participants had important insights on aspects of the AI intervention to be retained and refined in future evaluations. Elements to be retained include the 3-hour duration of each AI session, the close spacing of the AI sessions (preferably two sessions per week), the methods used by the Process Facilitator to enhance participation and productivity (*e.g.*, individual, paired, and group activities; giving activities in advance; acknowledging issues and challenges; recording results real time; and providing synopses), the eclectic group mix, an internal-external facilitator partnership, and the development of a concrete action plan. The usefulness of a defined action plan may be particular to implementing the AI intervention in nursing, given the recognized culture of task completion and busyness [36,37], as highlighted by participants in this study. The action plan may have had an important function in providing role clarity, supported by participants not carrying forward their efforts beyond what was outlined in the plan or organising the desired follow-up session. Role clarity has been previously identified as an important influencer of nurses' success as champions of evidence-based pain practices [38].

Refinements include those important to enhancing the clinical utility and sustainability of the AI intervention. Participants suggested adding an AI session to accommodate the potential need for more time, given the excess demands of the Dream and Design phases. Alternately, these AI sessions could be streamlined by using creative communication solutions, like online discussion forums. For example, designing and reviewing individual Provocative Propositions could be conducted away from the group sessions and posted online without impacting the collaborative nature of the AI sessions. Only one 3-hour AI session should be offered per day to respect the intensive nature of the activities, and a facilitator-led follow-up session and the identification and training of local champions should be included in the action plan to enhance sustainability. The intervention manual requires modifications to specify how local champions of the AI intervention would be selected, their roles and responsibilities, and the content of their training. In the future, the reality of fluctuating participation will be built into the AI sessions [39]. Given the inclusive spirit of AI [11] and the importance of buy-in from those in leadership positions evidenced in this study and others [34,40,41], future implementations will include all individuals interested in participating; however, a core group who maintain consistent attendance could be charged with championing the implementation of the action plan [39], as occurred naturally in this study with the staff nurses. Given the likely importance of interprofessional collaboration to implementing evidence in practice [42] and high-quality pain management practices [43], group membership needs to be expanded to interprofessional members of the healthcare team. Lastly, monetary compensation should be decreased to increase the clinical utility of the intervention. This alteration could be balanced by obtaining buy-in from high-level management to release staff to attend the AI sessions and implement the action plan based on the importance of developing evidence-based practices [34].

Given these refinements and remaining questions about conducting the AI intervention in different contexts, especially those that may seem less conducive to AI and the implementation of pain management evidence in practice than that in this study, it is vital that a process evaluation be included in a larger multisite effectiveness study. Research questions on process should focus on the feasibility of finding interested and qualified facilitators in other contexts; the impact of variably qualified facilitators on the fidelity of the intervention; the acceptability and feasibility of identifying and training local champions to ultimately assume sustained facilitator roles; the dose of the AI intervention required to produce the expected effects if variable levels of participation are allowed; the impact of decreasing monetary compensation on issues like recruitment and levels of participation; and the acceptability and feasibility of opening participation to the interprofessional team, given the potential challenges associated with engaging group members with different professional demands, priorities, and interests.

### Limitations

First, this case study involved one unit and the results are therefore specific to this group of participants, in this particular context; however, participants provided contextual descriptions (*i.e.*, culture, resources) that support the transferability of the results by allowing others to compare the congruence of this setting with their own [44]. Second, there was the potential for social desirability [45] to influence participants' accounts of their experience with the AI intervention based on professional expectations around evidence-based practice, a context attentive to excellence in pain management, and possible inclinations to report on a positively focused intervention in a positive way. Efforts were made to minimize the effects of this influence by informing participants that their responses would be kept confidential, there were no right or wrong answers [45], and both positive and negative feedback were important to refining the AI intervention. Third, participants were asked to give retrospective accounts of their experiences with the AI intervention. Despite this potential limitation, participants provided rich and detailed descriptions of their experiences that corroborated with each other in terms of the more factual aspects (*e.g.*, structure of the AI sessions, timing of action plan implementation). Last, there was the possibility for researcher influence during the qualitative analysis consequent to the role of the lead author as a facilitator of the AI sessions. A reflexive journal was maintained to capture assumptions, and, although the lead author was aware that she had an underlying desire for the intervention to succeed, she also had an equal interest in learning about areas for improvement for future research.

## Summary

The innovative use of AI as a KT intervention applied to a clinical issue in an inpatient health care setting was reported in this study. AI was an acceptable and feasible KT intervention that was implemented with high fidelity. Given these encouraging results, a larger multisite evaluation of the AI intervention is warranted. The AI intervention requires minor revisions before it is applied in future research, particularly to enhance sustainability. Future studies need to include process evaluations to determine the acceptability, fidelity, and feasibility of the modified AI intervention in other contexts and for other clinical areas.

## Competing interests

The authors declare that they have no competing interests.

## Authors' contributions

This work was derived from TK's doctoral thesis. TK conceived of and developed the study, conducted the quantitative data collection, delivered the AI intervention, analysed the data, and interpreted the findings. BS (supervisor), KS, SS, and JWW comprised the thesis committee and contributed to all aspects of study development and interpretation. TK drafted the manuscript and all authors commented. All authors read and approved the final manuscript.
